# Antinociceptive Effect of Rat D-Serine Racemase Inhibitors, L-Serine-O-Sulfate, and L-Erythro-3-Hydroxyaspartate in an Arthritic Pain Model

**DOI:** 10.1100/2012/279147

**Published:** 2012-03-12

**Authors:** Claudio Laurido, Alejandro Hernández, Teresa Pelissier, Luis Constandil

**Affiliations:** ^1^Laboratorio de Neurobiología, Facultad de Química y Biología, Universidad de Santiago de Chile, Santiago 9170022, Chile; ^2^Centro para el Desarrollo de la Nanociencia y la Nanotecnología, CEDENNA, FB-0807, Línea No. 8, Chile; ^3^Programa de Farmacología Molecular y Clínica, Facultad de Medicina, Universidad de Chile, Santiago 8380453, Chile

## Abstract

N-methyl-D-aspartic acid receptor (NMDAr) activation requires the presence of D-serine, synthesized from L-serine by a pyridoxal 5′-phosphate-dependent serine racemase (SR). D-serine levels can be lowered by inhibiting the racemization of L-serine. L-serine-O-sulfate (LSOS) and L-erythro-3-hydroxyaspartate (LEHA), among others, have proven to be effective in reducing the D-serine levels in culture cells. It is tempting then to try these compounds in their effectiveness to decrease nociceptive levels in rat arthritic pain. We measured the C-reflex paradigm and wind-up potentiation in the presence of intrathecally injected LSOS (100 *μ*g/10 *μ*L) and LEHA (100 *μ*g/10 *μ*L) in normal and monoarthritic rats. Both compounds decreased the wind-up activity in normal and monoarthritic rats. Accordingly, all the antinociceptive effects were abolished when 300 *μ*g/10 *μ*L of D-serine were injected intrathecally. Since no *in vivo* results have been presented so far, this constitutes the first evidence that SR inhibitions lower the D-serine levels, thus decreasing the NMDAr activity and the consequent development and maintenance of chronic pain.

## 1. Introduction

D-serine, a coagonist of the glycine site of the NMDAr, have been involved in many neurodegenerative disorders, in which pathological overactivation of the receptor results in neurotoxicity, as revealed by studies performed in cell cultures and observed in the Alzheimer disease. The overactivation of the NMDAr contributes also to acute disorders as ischemia [1]. In the *in vitro* studies, D-serine induced cytotoxicity in hippocampal slice cultures, since the removal of endogenous D-serine completely abolishes NMDA neurotoxicity. In serine racemase KO mice (SR-KO), around 90% decrease in forebrain D-serine content has been observed, and in parallel, a reduced neurotoxicity induced by both NMDA and *β*-Amyloid1-42 forebrain SR-KO mice injection was found. The former strongly suggest that SR is the responsible for the major D-serine production (Ran Inoue). Microglia culture cells exposed to amyloid *β*-peptide as a proinflammatory stimuli elicited a release of glutamate. This activated microglia express SR, and thereby release D-serine, contributing to the neurotoxicity found in inflammatory conditions in the brain, and also in the Alzheimer's disease where SR RNA was elevated [2]. Regarding D-serine degradation, it is performed by the D-amino acid oxidase, thus contributing to the D-serine homeostasis [3]. Another mechanism proposed to be responsible of D-serine modulation is gated by NMDAr activation, promoting the translocation of SR to the plasma membrane, thus reducing enzyme activity [4]. In brain areas where D-amino acid oxidase is poorly expressed or absent, (forebrain areas) degradation of D-serine is accomplished by a self-degradation mechanism by the *α*, *β*-elimination of water [5]. A cell line (HEK293) expressing mouse serine racemase was used to test L-serine-O-sulfate as an alternative substrate. The authors found that the degradation of L-serine-O-sulfate (to pyruvate and ammonia) was two or three orders of magnitude faster than the L-serine racemization. Also, they demonstrated the pharmacological inhibition of D-serine synthesis in cultured astrocytes, thus providing a strategy to selectively decrease NMDAr activation [6].

## 2. Material and Methods

### 2.1. Animals

Male Sprague-Dawley rats weighing 280–320 g were utilized in the present study. The ethical standards and guidelines for researchers on experimental animal pain were according to the Ethical Committee from the University of Santiago of Chile and the Committee for Research in Ethical Issues of the IASP, 1983 [7].

### 2.2. Rat Monoarthritic Model

For the monoarthritic rat model, complete Freund's adjuvant was prepared by adding 60 mg of killed *Mycobacterium butyricum* (Difco Laboratories, USA) bacteria to a mixture of 6 mL paraffin oil, 4 mL of NaCl 0.9% and 1 mL of Tween 80. The mixed suspension was then autoclaved for 20 min at 120°C., to rupture the mycobacterium cell walls. In order to induce the monoarthritis, the rats were injected 0.05 mL of the complete Freund's adjuvant into the left tibiotarsal joint under a brief halothane anesthesia. Control rats were given 0.05 mL of the vehicle used to suspend mycobacteria [8].

### 2.3. Drug Administration

Chemicals and their sources were as follows: L-serine-O-sulfate (LSOS) from Sigma, L-erythro-3-hydroxyaspartate (LEHA) from Wako Chem. Both LSOS and LEHA were dissolved in saline (0.9% NaCl) and injected intrathecally (i.t.) 100 *μ*g/10 *μ*L for LSOS and 100 *μ*g/10 *μ*L for LEHLA. D-serine was dissolved in saline and injected one bolus of 300 *μ*g/10 *μ*L.

### 2.4. Electrophysiological Evaluations

#### 2.4.1. C-Fiber Reflex Nociceptive Test

The C-fiber reflex will be evaluated in the normal and monoarthritic paw using urethane (1.2 g/kg i.p.) anesthetized animals. The C fibers will be stimulated by means of supramaximal electrical shocks applied on the 4th and 5th toes territory innervated by the sural nerve, by means of two stainless steel needles. Then, with 0.1 Hz stimulation with intensity of two times, the electromyographic threshold will be maintained through the whole experiment. The electromyographic activity will be registered from the *biceps femoris* ipsilateral [9]. The electromyographic responses will be loaded to a computer provided with a digital to analog converter, and *ad hoc* software and the absolute value of the integrated response (expressed in Volt per second) taken in a time window opened between 150 to 450 ms after the stimulus (time zero) will constitute the C-reflex response. Animals with simulated arthritis will serve as controls. This C-fiber activated reflex is equivalent to the R-III reflex recorded in man, representing a direct proportionality among subjective pain perception and the electromyographic intensity.

#### 2.4.2. Spinal Wind-Up

The same initial C-reflex protocol is followed here. In order to evoke the synaptic potentiation phenomena or wind-up, ten 1.0 Hz stimuli will be applied. This initial testing will be the control. For all the cases, only values showing increment in the integral will be used. It happens usually between the third to eighth stimuli. In order to quantify the wind-up effect, we define the term percent of algesia as

(1) $$\%\;\text{Algesia}=\left[\left(\frac{\text{WU}*100}{{\text{WUC}}_{t=0}}\right)-100\right],$$

where WU means the wind-up value of the normal and monoarthritic rats in different times; WUC_t=0_ means the wind-up value of the normal rats at time zero. With this equation, points showing positive values signify hyperalgesia (due to the monoarthritis) and negative values meaning analgesia.

### 2.5. Analysis of Results

Results were expressed as means ± SEM., *N* = 6 rats in all groups. *P* < 0.05 according to two way ANOVA.

## 3. Results

We have studied the effect of two compounds that were able to reduce the activity of the serine racemase *in vitro* or in cell cultures. Among the most effective competitive inhibitors are small amino and dicarboxylic acids like EBHA with a *K*_*i*_ = 43 *μ*M and malonic acid (*K*_*i*_ = 71 *μ*M), among others. Unfortunately, the last was uneffective in our studies up to 10 *μ*g/10 *μ*L i.t. (results not shown). L-serine-O-sulfate is one of the aminoacids able to inhibit the serine racemase activity, with D-cysteine and L-lysine (not tested in this work). Other aminoacids were proven to be ineffective [5].

### 3.1. Effect of LSOS and LEHA on C-Reflex Activity

**Figure 1:**
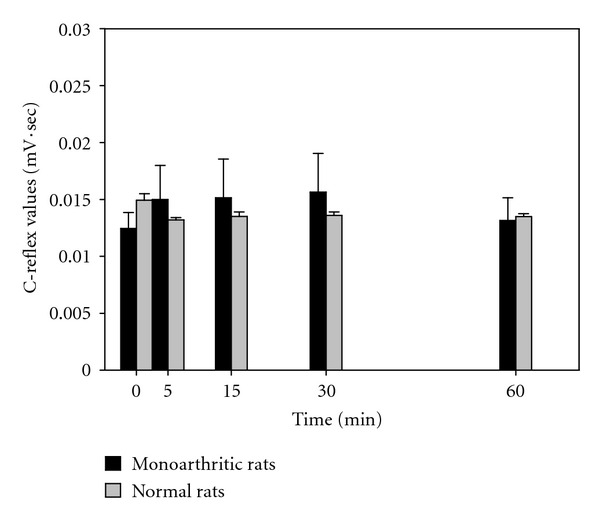
Effect of LSOS on the spinal C-reflex. The figure shows the C-reflex response after the application of 100 *μ*g/10 *μ*L of LSOS i.t. at time zero. Both normal and monoarthritic rat responses are not modified by the LSOS treatment, indicating that LSOS is not involved in the C-reflex response. (Values are mean ± SE, *n* = 6 rats each group).

**Figure 2:**
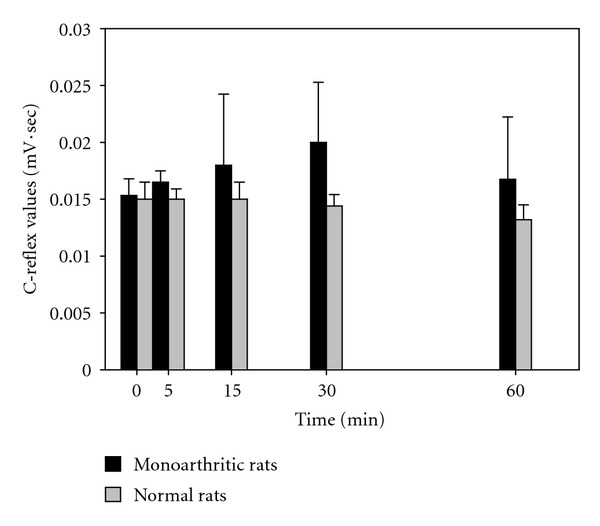
Effect of LEHA on the spinal C-reflex. The figure shows the C-reflex response after the application of 100 *μ*g/10 *μ*L of LEHA i.t. at time zero. Both normal and monoarthritic rat responses are not modified by the LSOS treatment, indicating that LEHA is not involved in the C-reflex response. (Values are mean ± SE, *n* = 6 rats each group).

Figure 1 shows the C-reflex response after the application of LSOS (100 *μ*g/10 *μ*L) at time zero and LEHA (100 *μ*g/10 *μ*L) at time zero (Figure 2). It can be observed that the C-response either in normal or monoarthritic rats is constant and the C-reflex paradigm is unaffected by the both treatments. The former due probably to the fact that the response at lower frequencies (enough to evoke the C-reflex) of the sural nerve electrical stimulation, involves the activation of AMPA/Kainate receptors and not the NMDAr, blocked by magnesium ions [10].

### 3.2. Effect of LSOS and LEHA on Wind-Up Activity

**Figure 3:**
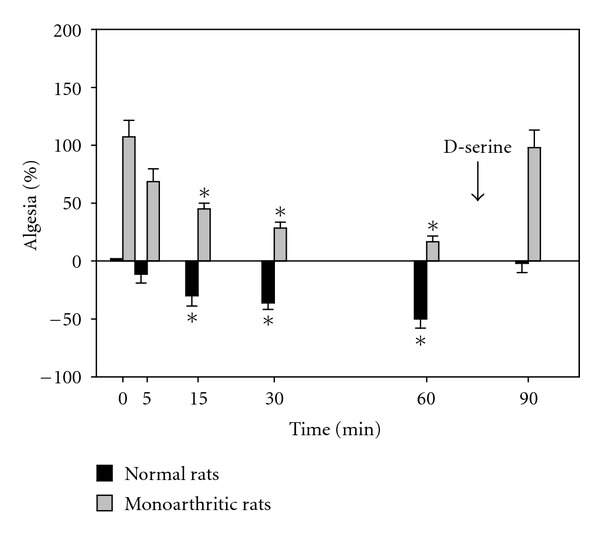
Effect of LSOS on spinal wind-up. The figure shows the effect of LSOS i.t. on spinal wind-up at time zero. There is hyperalgesia in the monoarthritic rats, due to his arthritic condition. The observed tendency is to diminish this hyperalgesia, being statistically significant from time 15 minutes (*P < 0.05, Two-way ANOVA). On the other hand, normal rats present analgesia, being statistically significant from time 15 minutes reaching a value of around 50% at time 60 minutes with respect to the control animals. At 75 min, D-serine (300 *μ*g/10 *μ*L) is injected i.t., and at 90 min, both the hyperalgesia in monoarthritic rats and analgesia in normal rats return to the original values found at time zero. (Values are mean ± SE, *n* = 6 rats each group).

Both compounds were able to decrease the wind-up activity in normal and monoarthritic rats. There was a significative lowering in the hyperalgesia produced in the monoarthritic rats and a progressive return to a normal condition. In normal rats, both compounds acted as antinociceptive. In Figure 3, the effect of LSOS is depicted. At time zero, the hyperalgesia produced by the monoarthritic condition can be seen. The application of LSOS diminishes the hyperalgesia and becomes significative from time 15 minutes. Nearly 75% of the hyperalgesia decrement is achieved with the LSOS treatment at time 60 minutes. The normal rats showed analgesia with respect to time zero becoming statistically significant from time 15 minutes. Nearly 50% of decrement is achieved at time 60 minutes. At 75 min, D-serine (300 *μ*g/10 *μ*L) is added i.t. Then, at 90 min, it can be observed that D-serine restitutes the hyperalgesia in monoarthritic rats and the analgesia in normal rats. Values observed are not different from those obtained at time zero.

**Figure 4:**
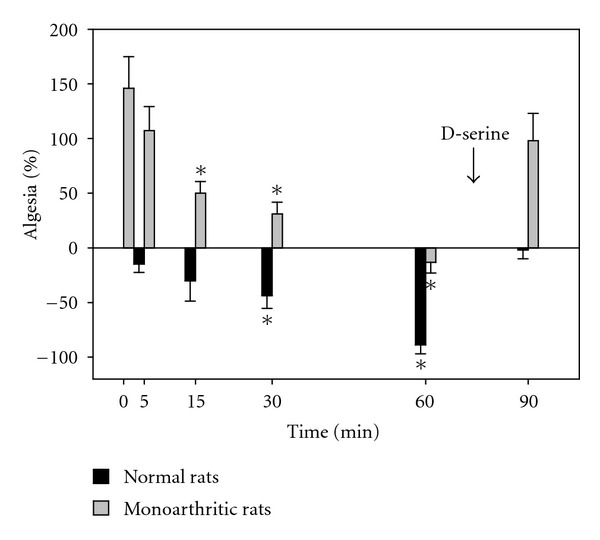
Effect of LEHA on spinal wind-up. The figure shows the effect of LEHA i.t. on spinal wind-up at time zero. There is hyperalgesia in the monoarthritic rats due to his arthritic condition. The observed tendency is to diminish this hyperalgesia, being statistically significative from time 15 minutes (*P < 0.05, two-way ANOVA). At 60 min, the hyperalgesia turns into analgesia, indicating that this compound is more efficient than LSOS. On the other hand, normal rats present analgesia, being statistically significant from 30 min reaching a value of around 75% at 60 min with respect to the control animals. At 75 min, D-serine (300 *μ*g/10 *μ*L) is injected i.t., and at 90 min, both the hyperalgesia in monoarthritic rats and analgesia in normal rats return to the original values found at time zero. (Values are mean ± SE, *n* = 6 rats each group).

The effect of LEHA is shown in Figure 4. As can be seen, the results are nearly the same. In order to be sure that both groups of rats behave the same, the hyperalgesia observed in the monoarthritic rats at time zero was not significantly different from the hyperalgesia of the monoarthritic rats treated with LSOS. The hyperalgesia is diminished with the LEHA treatment and becomes significant from time 15 minutes. In this case, the effect is important achieving more than 100% of hyperalgesia reduction, since at time 60 minutes, the hyperalgesia becomes analgesia. The i.t. injection of D-serine (300 *μ*g/10 *μ*L) at 75 min restitutes the values to the zero time.

## 4. Discussion

Arthritic pain constitutes a serious health problem in our society. NMDAr plays an important role in the development and maintenance of chronic pain. This receptor is also involved in several other processes like learning, behavior, and long-term synaptic plasticity in the hippocampus. At present, among the many strategies utilized for the treatment of chronic pain, the use of nonsteroidal anti-inflammatory drugs [11] and electroacupuncture [12] arises as promising tools. Both, unfortunately, are presenting serious drawbacks. The first, in long-term treatments, causes the presence of gastrointestinal ulcers or kidney damage. The last leads to the poorly understood mechanisms leading to electroacupuncture analgesia. Both NMDA receptor blockade or inactivation often results in undesirable side effects; for example, if the blockade is too specific, then hallucinations is one of the prominent side effect found. On the other side, NMDA receptor overactivation produces neuronal death leading to brain damage [13]. Since the only intrinsic source known so far of D-serine synthesis in the organism is the racemization of L-serine produced by the D-serine racemase, this pathway is a key process for the cell to obtain this amino acid. Then, any effort to lower D-serine concentration in nociceptive synapses will result in the inhibition of the NMDAr-mediated neuronal activity and thus producing antinociception. The treatment with these two compounds reduced the hyperalgesia in the monoarthritic rats, and with a proper adjustment of dosage, we expect the complete suppression of pain in this animal pain model. It is interesting that only the wind-up was affected by the treatment, since the C-reflex was not affected indicating that only the NMDAr are involved in the antinociceptive effect described. We tested also malonic acid but without success, even with a *K*_*i*_ = 71 *μ*M, in the order of magnitude of EBHA. Free malonate is present at a high concentration in the rat brain (192 nmol/g wet weight) [14], but the reason why it did not present any effect on serine racemase remains to be elucidated. Regarding the toxicity of these compounds, we injected 500 *μ*g/10 *μ*L i.t. of both compounds by means of a brief isoflurane (5% in 100% O_2_) anesthesia and followed up the behavior of the rats, both normal and monoarthritic, for 5 days. The idea was to compare the rat behavior with the control rats by visual observation. Since it was undistinguishable from the control rats, we assume that the compounds at the concentration utilized were innocuous to the rats. Finally, the fact that the drugs were injected i.t. and not systemically and at lower levels of the spinal cord (L5-L6), the possibility of interference of undesirable side effects (learning, behavioral abnormalities) at the supraspinal level is abolished. As a conclusion, we can say that rheumatoid arthritis remains a major health problem worldwide, with a prevalence that may amount to one case per 100 people depending on the geographical area of the world considered. Among other major impairing health problems associated with rheumatoid arthritis, pain emerges as the most commonly reported and prevalent debilitating condition, but current therapies are still suboptimal. The use of serine racemase inhibitors might constitute a new tool for the treatment of this condition and applicable to other chronic pain syndromes.
